# Anisotropic Properties of Polylactic acid–carbon Fiber Composites Prepared by Droplet spray Additive Manufacturing

**DOI:** 10.3390/ma12040669

**Published:** 2019-02-23

**Authors:** Yongfeng Li, Qingjun Ding, Hongyuan Zhao, Tingting Wu, Mingming Zhang, Yaqi Zhang

**Affiliations:** 1 School of Mechanical and Electrical Engineering, Henan Institute of Science and Technology, East of Hualan Road, Xinxiang 453003, China; hongyuanzhao@126.com (H.Z.); wtingtingwu@163.com (T.W.); zmmhqm@163.com (M.Z.); zhangyaqi_1988@163.com (Y.Z.); 2 State Key Laboratory of Mechanics and Control of Mechanical Structures, Nanjing University of Aeronautics and Astronautics, Nanjing 210001, China

**Keywords:** droplet spray, additive manufacturing, anisotropy, carbon fiber

## Abstract

Anisotropic materials are important functional materials in many fields. The use of these materials is currently being expanded through the rapid development of additive manufacturing. However, there is still no universal method for fabricating two-dimensional anisotropic polymer composites. Here, polylactic acid–carbon fiber composites were prepared using the droplet spray method, and their mechanical and friction properties were studied. The tensile strength in the X–Y plane perpendicular to the direction of the droplet spray was significantly higher than that in the direction of droplet ejection. Similar trends were observed for the elongation at breaking and the impact strength. The friction coefficient was smallest in the X–Y plane. Scanning electron microscopy showed that carbon fibers were oriented in the X–Y plane, which enhanced the mechanical and friction properties in this plane.

## 1. Introduction

Anisotropy of polymer composites in fillers results in directional macroscopic properties [1,2]. One- and two-dimensional oriented anisotropic polymer composites have excellent properties, such as conductivity, thermal conductivity, magnetism, permeability and mechanical strengths, etc. [3,4,5]. They are widely used in biomedicine, electromagnetic shielding, stealth technology and aircraft fuselage materials [6,7]. In two-phase polymer composites, if one of the phases (e.g., the phase with high thermal conductivity) is uniformly dispersed without any orientation, the thermal conductivity is low at relatively low filler content. When the filler content is very high, an ideal thermal conductivity can be achieved; however, this might come with a loss in other material properties and/or an increase in cost. By contrast, a high thermal conductivity at low filler content can be achieved if the phase with high thermal conductivity is parallel to the direction of heat flow and forms a penetrating structure. The use of such anisotropic structures is an effective way to reduce the filler content while retaining the properties of the polymer filler [8].

Common methods for preparing one-dimensional oriented anisotropic polymer composites are shear force orientation [9,10], magnetic field orientation [11,12], electric field orientation [13,14,15], pre-orientation, thermal gradient self-assembly orientation of copolymers [16] and solvent evaporation-driven orientation [17]. By comparison, there are relatively few preparation methods for two-dimensional oriented polymer composites, which can usually only be prepared using fiber cascades and other special methods. 

For example, Sohn and Seo [16] obtained a lamellar microphase structure of PS-b-P4VP block copolymer by spin coating and annealing field treatment. Prefabricated fiber-liquid resin casting has also used to prepare one-dimensional and two-dimensional oriented polymer composites for engineering applications [17], typical FRP has been widely used, such as epoxy resin composites filled with glass fiber filled, Boron fiber filled phenolic resin.

Compared with the conventional methods described above, additive manufacturing—which generates three-dimensional entities by adding materials layer-by-layer through continuous physical stacking—has several advantages [18,19]. The main advantages of the additive manufacturing method are as follows: 1. It has a shorter production cycle. 2. It can manufacture complex parts without the limitation of traditional processing. 3. In the manufacturing process, it can save raw materials, and there is no waste nor recycling. 4. It has precise entity replication capability. Easir Arafat Papon et al. [20] studied the effects of carbon fiber reinforcement, nozzle geometry and bead lay-up orientations in fracture properties, void contents, and interfacial bonding. The results showed that there were significant improvements in fracture toughness and fracture energy for CF/PLA composites over pure PLA. Ning et al. [21] produced one- and two-dimensional anisotropic composites using two nozzles on the additive manufacturing machine to output long fibers and resins; however, the infiltration of the melt resin on the fibers was poor. Tekinalp et al. [22] blended resins and fibers with an average length of 3.2 mm and extruded them from nozzles with a diameter of 0.5 mm. Quan et al. [23,24] studied the microstructure of additively manufactured ABS resin composites, which they fabricated by adding short fibers, and continuous carbon fibers as the skeleton. They found that the short fibers led to a certain degree of one-dimensional orientation. They also analyzed the mechanism of orientation driven by shear force. However, despite these efforts, there is no universal preparation method for two-dimensional oriented anisotropic polymer composites.

In this work, two-dimensional oriented anisotropic polymer composites were fabricated, which were based on deformation and shear-force driving of droplets during impact-spreading using droplet-spray additive manufacturing. The obvious anisotropy in the mechanical properties and friction property was investigated.

## 2. Materials and Methods 

### 2.1. Materials

**Figure 1:**
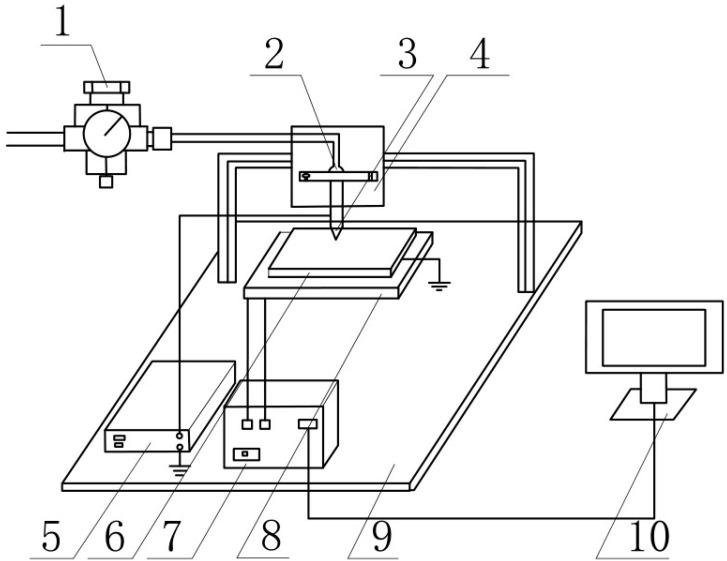
Schematic diagram of the printing system.

Raw materials were used as follows: Polylactic acid (PLA) from NatureWorks, Minnetonka, MN, USA (product name: 2003D). Dichloromethane (≥99.8%) and carbon fiber (WD300) were obtained from Aladdin Reagent Co., Ltd. (Shanghai, China). The carbon fibers had a diameter is 7 μm with an average aspect ratio of 5, and the original fiber had an anti-extension modulus of 230GPa and a tensile strength of 3.8 GPa. The experimental setup was a self-built spray printing system, as shown in Figure 1. Number 1 is the pneumatic device, number 2 is the cartridge, number 3 the spray needle, number 4 is the Z-axis manual adjusting table, number 5 is a high-voltage power supply, number 6 is the base board, number 7 is the control system, number 8 the X–Y electric platform, number 9 is the workbench and number 10 the computer. The needleused was a 30G stainless steel dispensing needle with an inner diameter of 0.7 mm.

The surface of the carbon fibers was treated with silane coupling agent KH-550. The sol concentration has a substantial impact on film formation; if the concentration is too high, the nozzle will be blocked easily, which makes the experiment less operable; if the concentration is too low, carbon fibers settle readily. Therefore, after analysis of several initial experiments, a PLA concentration of 0.3 mol/L was selected, and carbon fibers (10 wt% relative to the weight of polylactic acid) were added to the solution. The solution was then stirred magnetically for 2 h, followed by ultrasonic dispersion for 20 min at a frequency of 40 KHz and power of 250 W. The microdroplet injection system was then opened and the parameters were adjusted. The distance between the nozzle and the substrate was 0.6 mm, the pressure was 1.2 kPa and the voltage was 1.0 kV.

**Figure 2:**
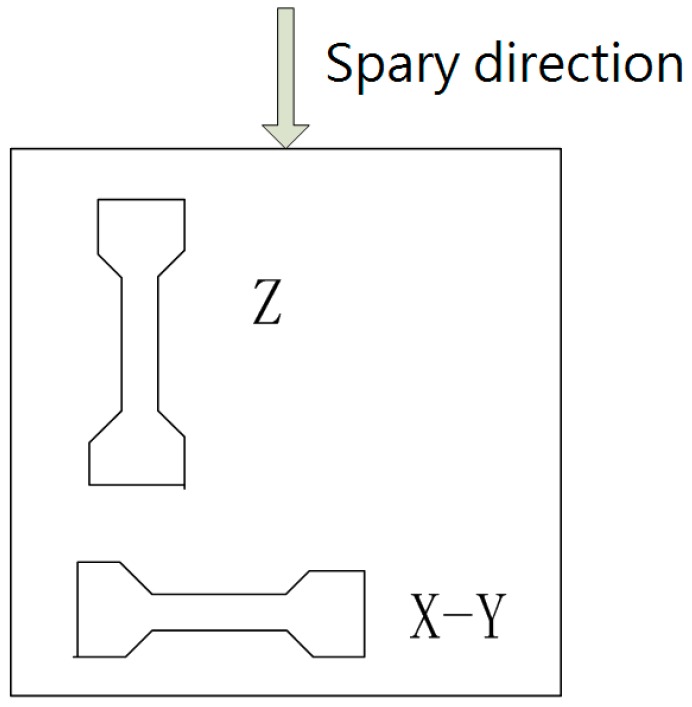
Diagram of the specimen.

The horizontal plane perpendicular to the direction of injection was defined as the X–Y plane. The plane parallel to the direction of injection was defined as the Z plane. These orientations are shown in Figure 2.

### 2.2. Performance Testing Methods

#### 2.2.1. Mechanical Property Testing

The tensile strength and elongation upon breaking of the films were tested using a WDW-5000N tensile tester (Shanghai Huai Instrument Equipment Co., Ltd., Shanghai, China). According to GB/T 1041-2008, the samples were cut into dumbbell shapes. To ensure the accuracy of the tensile results, five samples of each specimen were prepared. The two extreme values were removed and the average value was taken as the final result. Similarly, the bending properties were tested according to ASTM D790-2003, and the impact properties were tested using a TY-4020-15Jimpact testing machine (Jiangsu Tianyuan Testing Equipment Co., Ltd., Yangzhou, China) according to GB/T 1043-2008.

#### 2.2.2. Friction and Wear Testing

The orientation of carbon fibers has a significant effect on the friction and wear properties [25,26]. In this work, the tribological properties of specimens under dry friction were tested using an HSR-2M friction and wear tester. The sensor size was 1000 gf, the load was 2 N, the motor speed was 200 rpm, and the friction time was 30 min. Silicon nitride spheres (4 mm diameter) were used as friction coupling.

#### 2.2.3. SEM Characterization

The samples were scanned using a Hitachi S-4800 scanning electron microscope (SEM, Hitachi S-4800, Tokyo, Japan) under ultrahigh pressure (EHT 5000V). A conductive adhesive spray was used to attach the sample to the base of the SEM.

## 3. Results and Discussion

### 3.1. Mechanical Properties

**Figure 3:**
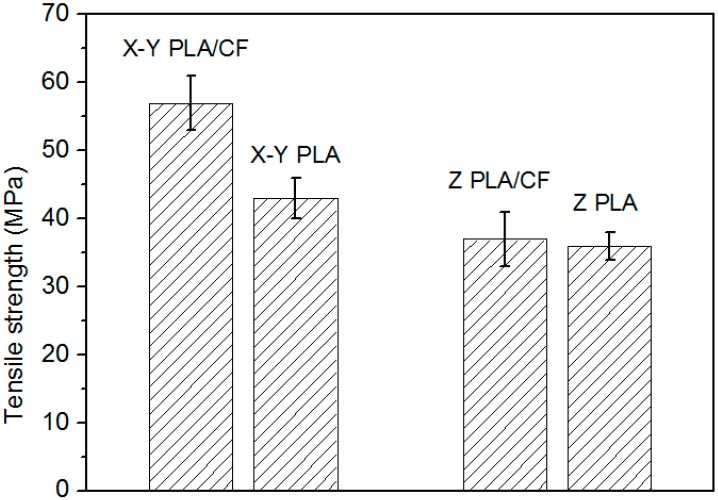
Tensile strength of the polylactic acid–carbon fiber (PLA–CF) specimens.

The tensile strength of the sample is shown in Figure 3. The tensile strength of PLA without carbon fibers differs only slightly (10%–20%) between the X–Y PLA and Z–PLA. The reason for this is that the bonding strength between layers is weaker than the strength of the PLA body, and the solvent blurs the interface between layers. This results in lower anisotropy than is commonly observed in 3D printing. For example, the difference in tensile strength in different directions of FDM (Fused Deposition Modeling) printing is generally more than 50% [27]. When carbon fibers were added to the PLA, the tensile strength in the X–Y plane was about 50% higher than that in the Z–plane. In addition, the carbon fibers change the tensile properties of the specimen. In the X–Y plane, the addition of carbon fibers increases the tensile strength by about 40%, whereas the strength in the Z–direction hardly changes. Moreover, the test standard deviation of the samples with carbon fibers is larger than that of pure PLA. This is mainly because of insufficiently uniform dispersion of fibers and their effect on the interfacial bond strength between layers.

**Figure 4:**
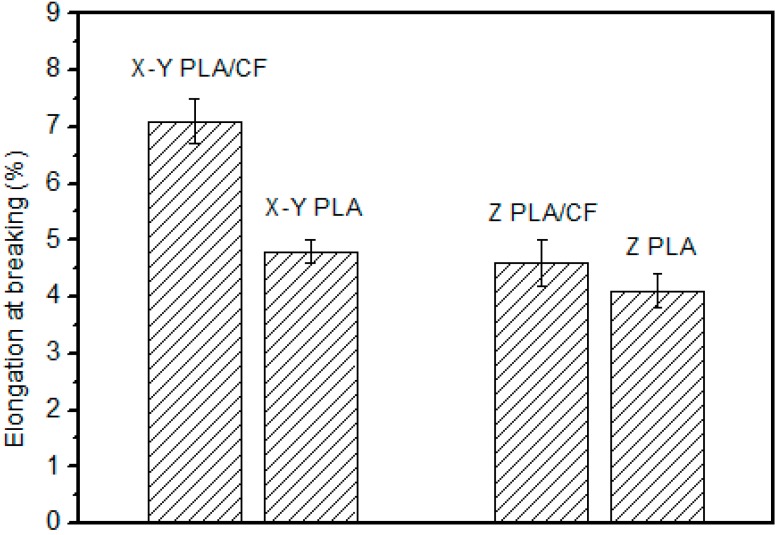
At breaking of the polylactic acid–carbon fiber (PLA–CF) specimens.

Figure 4 shows the elongation upon breaking of the specimen. The trend among specimens is similar to that for the tensile strength. The elongation at breaking of PLA without carbon fibers is 20% larger in the X–Y plane than in the Z–plane, which is mainly because of the weak bonding force between layers. By contrast, in the presence of carbon fibers, there is a large difference in elongation between the two directions.

**Figure 5:**
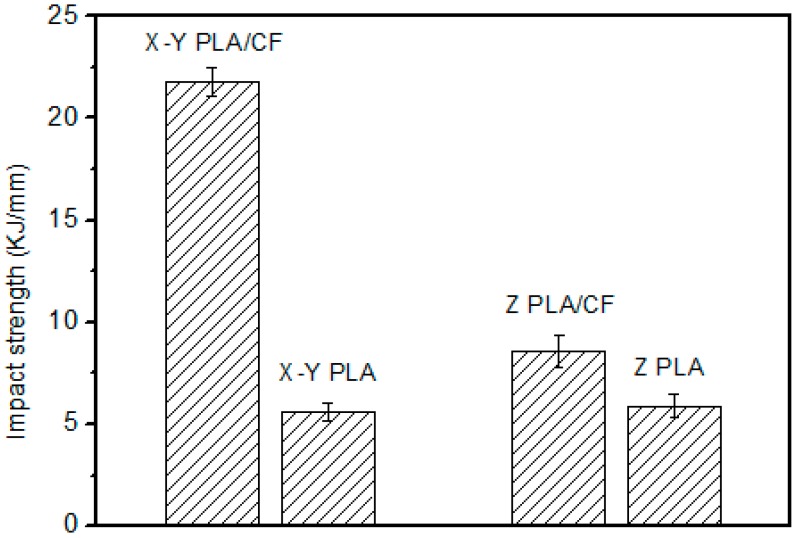
Strength of the polylactic acid–carbon fiber (PLA–CF) specimens.

The impact strength of the different specimens is shown in Figure 5. The general trend among specimens is similar to that of the tensile strength, but much more pronounced. The impact strength was 300% higher in the X–Y plane with carbon fibers than in the Z–plane without carbon fibers. This is because the carbon fibers confer impact resistance of PLA and the effect of fiber orientation. The impact strength in the X–Y plane with carbon fibers is about 140% higher than that in the Z–plane of the specimens with carbon fibers. This is because of the fiber orientation in the specimens. The impact strength in the Z–plane of specimens with carbon fibers is about 50% higher than that without carbon fibers; the impact strength of specimens without carbon fibers differs only slightly between the two directions.

### 3.2. Friction Properties

**Figure 6:**
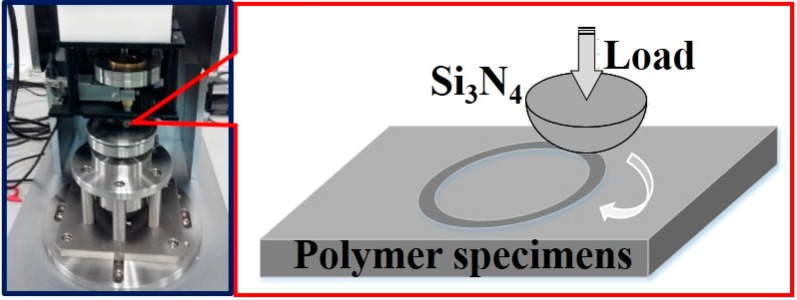
Equipment picture and contact schematic diagram for measuring friction.

The friction and wear tests of PLA composites sliding against an Si_3_N_4_ ball were evaluated on a ball-on-disk tribo-meter (HSR-2M, Zhongke Kaihua Technology Development Co., Lanzhou, China) at room temperature. The contact schematic diagram of the friction couple was shown in Figure 6. The Si_3_N_4_ ball, as a counterpart, was 4 mm in diameter. Before each test, the Si_3_N_4_ ball and the block samples were cleaned with cotton dipped in acetone. The friction coefficient was continuously recorded by an online data acquisition system attached to the tester.

**Figure 7:**
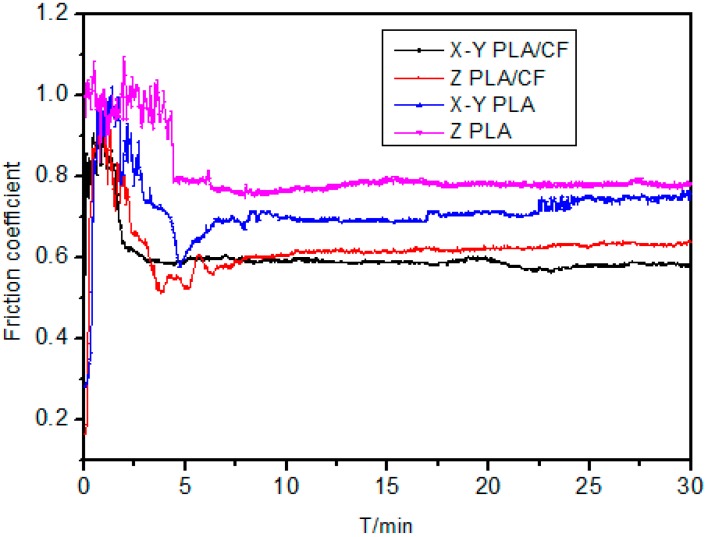
Friction coefficient of the polylactic acid–carbon fiber (PLA–CF) specimens.

The wear resistance of PLA is relatively poor, and there are few reports on the investigation of its friction and wear properties. To investigate the anisotropy of fibers after orientation in polylactic acid, the tribological properties of the fibers were measured. The friction coefficients (Figure 7) of the X–Y PLA–CF and Z-PLA–CF specimens are smaller than those of the X–Y PLA and Z-PLA specimens, which indicates that carbon fibers can reduce the friction coefficient of PLA; the friction coefficients of the X–Y PLA–CF specimens are smaller than those of the Z-PLA–CF specimens, and the friction coefficients of the X–Y PLA specimens are smaller than those of the Z-PLA specimens, indicating that microdroplet injection induces anisotropy.

### 3.3. SEM Results

**Figure 8:**
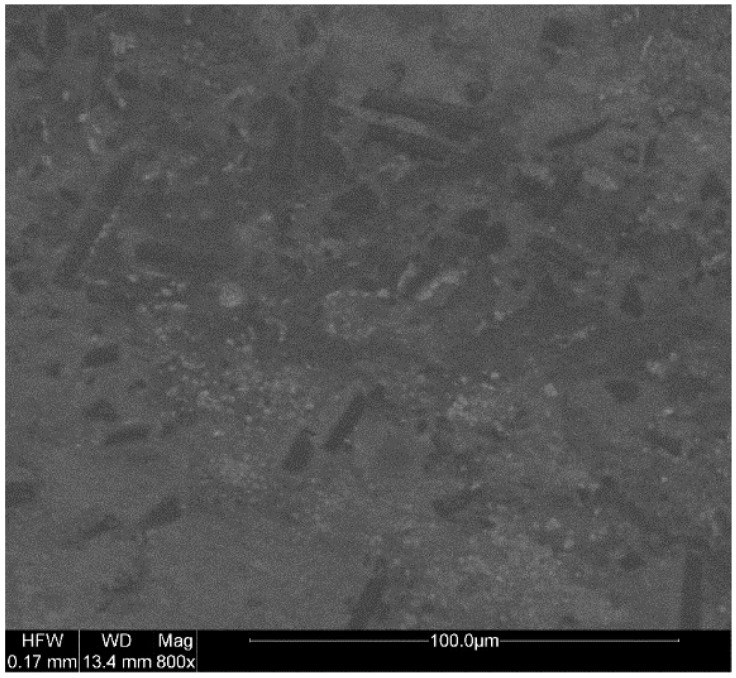
X–Y plane of the polylactic acid–carbon fiber specimen.

**Figure 9:**
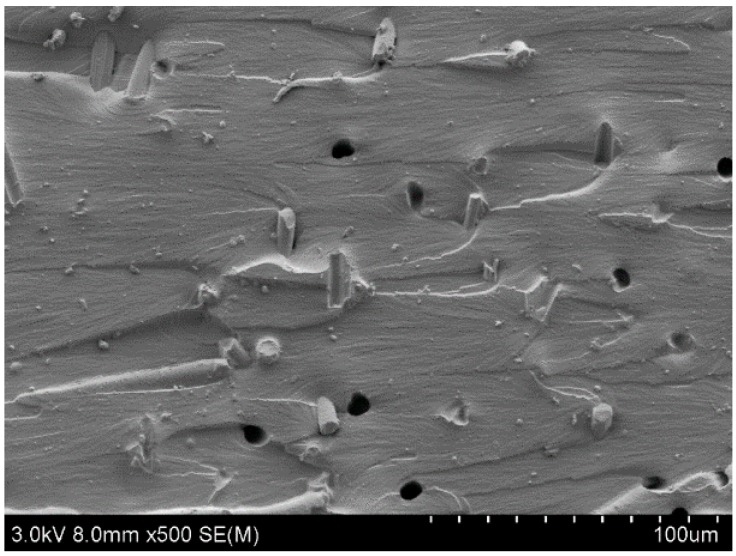
Tensile fracture cross-section of the X–Y polylactic acid–carbon fiber specimen.

Figure 8 shows a distribution diagram of short carbon fiber in the X–Y plane of the sample, and Figure 8 shows a cross-sectional image of the X–Y sample after the tensile test. The carbon fibers had a degree of orientation and were randomly distributed in the X–Y plane, as shown in Figure 8. Under tension (Figure 9), more carbon fibers are pulled out and are either oriented horizontally or inclined, which demonstrates that they are disorderly distributed in the plane perpendicular to the X–Y plane. Comparing Figure 8 and Figure 9, it can be considered that the long-diameter directions of carbon fibers are mostly in the X–Y plane, and are randomly distributed in the x–y plane, forming a two-dimensional anisotropy. Carbon fiber has high strength and good lubricating property. The two-dimensional anisotropy also partly explains the results of the above-mentioned mechanical and friction tests.

## 4. Conclusions

Polylactic acid–carbon fiber composites were fabricated by using the microdroplet spray method. The mechanical properties, frictional properties and the microscopic surface of the composites were tested. The main conclusions are as follows:

(1) The SEM images show that carbon fibers were oriented in the X–Y plane perpendicular to the direction of injection. 

(2) This orientation of carbon fiber enhanced the material anisotropy and resulted in superior, directional properties, such as strength.

(3) The carbon fiber reduces the coefficient of friction of the printed material, and the oriented carbon fiber further enhances this effect.

This work reports that it is critically important to understand the thermo-mechanical behaviors of 3D printing materials with carbon fiber, particularly anisotropy in some properties for the use of the emerging manufacturing technique, 3D printing, in a wide variety of engineering applications.
